# Two new species of *Ceratostomella* and *Pararamichloridium* (Sordariomycetes) from Guangdong, China, based on morphological and phylogenetic evidence

**DOI:** 10.3897/mycokeys.133.194192

**Published:** 2026-06-05

**Authors:** Weiqian Meng, Surapong Khuna, Tanapol Thitla, Ning Xie, Sinang Hongsanan

**Affiliations:** 1 Shenzhen Key Laboratory of Microbial Genetic Engineering, College of Life Sciences and Oceanography, Shenzhen University, Shenzhen 518060, China College of Life Sciences and Oceanography, Shenzhen University Shenzhen China

**Keywords:** Air-contaminated fungi, multi-locus phylogeny, novel species, rock-inhabiting fungi, Sordariomycetes, southern China

## Abstract

During a survey of fungal diversity in Guangdong Province, China, two fungal strains were obtained from air contaminants, and three fungal strains were recovered from rock surfaces. Morphological characterization combined with multi-locus phylogenetic analyses based on LSU, ITS, and SSU sequence data indicated that these strains belong to the genera *Ceratostomella* and *Pararamichloridium* within the class Sordariomycetes. Phylogenetic results showed that the strains form distinct and well-supported lineages, clearly separated from previously described species. Therefore, they are introduced here as two new species: *Ceratostomella guangdongensis***sp. nov**. and *Pararamichloridium purpureum***sp. nov**. Detailed descriptions, illustrations, and a phylogenetic tree are provided. This study highlights rock surfaces and air environments as valuable sources of previously undocumented fungal diversity.

## Introduction

Sordariomycetes is the second-largest class within the phylum Ascomycota, comprising several thousand species with diverse ecological functions and morphological characteristics (Wijayawardene et al. 2022). Members of this class are widely distributed across terrestrial, freshwater, and marine ecosystems and exhibit a broad range of ecological lifestyles, including saprotrophic, endophytic, pathogenic, and fungicolous modes of life (Maharachchikumbura et al. 2016; Zhang et al. 2023; Wang et al. 2024). Although considerable taxonomic progress has been achieved through integrative approaches combining morphological observations with molecular phylogenetic analyses, many lineages within this class remain insufficiently explored, particularly those inhabiting unusual or understudied environments.

Fungal communities associated with unconventional substrates, such as rock surfaces and airborne environments, remain poorly documented. Rock-associated fungi are increasingly recognized as an ecologically important group capable of surviving under extreme environmental conditions, including desiccation, temperature fluctuations, high solar radiation, and limited nutrient availability. These lithophilic or saxicolous fungi play significant roles in mineral weathering and contribute to the early stages of biofilm formation on rock surfaces (Liu et al. 2022). Similarly, airborne fungi represent a crucial dispersal phase in fungal life cycles, facilitating the distribution of propagules across diverse ecosystems (Abrego et al. 2024). Although numerous studies have investigated airborne fungal diversity in the context of environmental monitoring and plant pathology, many airborne isolates remain taxonomically unresolved due to the limited integration of detailed morphological characterization and robust phylogenetic analyses (Žilka et al. 2024). Consequently, studies focusing on fungi from these unconventional habitats are essential for improving our understanding of fungal diversity and evolutionary relationships.

Species of *Ceratostomella* have been reported from a variety of substrates, including decaying wood, soil, plant debris, and freshwater habitats across temperate to tropical regions (Réblová 2006; Maharachchikumbura et al. 2016). However, the taxonomy of this genus has undergone several revisions, and molecular phylogenetic studies have demonstrated that morphologically similar taxa previously assigned to *Ceratostomella* may represent multiple independent lineages within Sordariomycetes (Saccardo 1878; Réblová 2006; Réblová et al. 2024). As a result, the circumscription and species diversity of *Ceratostomella* remain incompletely resolved, highlighting the need for additional taxonomic studies integrating morphological characteristics with multilocus phylogenetic analyses.

*Pararamichloridium* was introduced by Crous et al. (2017) to accommodate two species, *P. livistonae* Crous (the type species) and *P. verrucosum* (V. Rao & de Hoog) Crous. Subsequently, three additional species, namely *P. aquisubtropicum* Jing Y. Zhang, Y.Z. Lu & K.D. Hyde, *P. caricicola* Crous, and *P. ouropretoense* Crous, were described from different substrates and geographic regions (Crous et al. 2018, 2025; Jayawardena et al. 2023). At present, five species are recognized within the genus. Members of *Pararamichloridium* have been reported mainly from plant-associated substrates and freshwater environments (Crous et al. 2017, 2018, 2025; Jayawardena et al. 2023). The genus is morphologically characterized by branched or unbranched, medium-brown, septate conidiophores; terminal and intercalary, medium-brown, denticulate conidiogenous cells; and solitary, hyaline, smooth, granular, aseptate, clavate conidia (Crous et al. 2017).

During an investigation of fungal diversity in Guangdong Province, China, five fungal strains were isolated from rock surfaces and air contaminants. Based on integrated evidence from detailed morphological observations and phylogenetic analyses of combined LSU, ITS, and SSU sequence data, these strains were confirmed to represent two distinct, previously undescribed species belonging to the genera *Ceratostomella* and *Pararamichloridium*. Notably, this study documents the first occurrence of *Ceratostomella* on rock substrates and *Pararamichloridium* isolated from air. In this study, we describe and illustrate these taxa as *Ceratostomella guangdongensis* sp. nov. and *Pararamichloridium purpureum* sp. nov.

## Materials and methods

### Sample collection

Rock samples were collected from Dananshan Mountain (22°30'06.7"N, 113°54'44.0"E), Shenzhen, China, in March 2025. Samples bearing flourishing black colonies were collected using a sterile chisel, placed in sterile plastic bags, and transported to the laboratory in an ice box. All samples were processed for fungal isolation immediately upon arrival. Air-contaminated fungi were isolated in 2025 from an uncleaned laboratory room at the College of Life Sciences and Oceanography, Shenzhen University (22°35'55.0"N, 113°59'21.0"E), Shenzhen, China.

### Fungal isolation

For rock-inhabiting fungi, isolation was performed following the method described by Thitla et al. (2023) with minor modifications. Rock samples were washed in 1% sodium hypochlorite for 10 min and rinsed five times with sterile distilled water. The samples were then pulverized, and the resulting rock powder was sprinkled onto 2% malt extract agar (**MEA**; Difco, Le Pont de Claix, France) and dichloran–rose bengal agar (**DRBC**; Difco, Le Pont de Claix, France) supplemented with chloramphenicol (100 ppm). Plates were incubated at 25 °C for four weeks and inspected daily. Colonies with dark pigmentation were transferred to fresh MEA plates for purification.

For air-contaminated fungi, isolation was conducted following the method of Sri-indrasutdhi et al. (2015) with minor modifications. Petri dishes containing potato dextrose agar (**PDA**; Difco™, Becton, Dickinson and Company, Maryland, USA) supplemented with streptomycin (0.5 mg/L) were exposed to indoor air at room temperature for 15 min. The plates were then sealed and incubated at 25 °C. Emerging fungal colonies were transferred to fresh PDA plates for purification.

All pure cultures were deposited and permanently preserved in a metabolically inactive state (dry culture) in the Herbarium of College of Life Science and Oceanography (**SZU**) and Culture Collection of Microbial Shenzhen University (**MBSZU**), Shenzhen University, China.

### Morphological characterization

Morphological characteristics were examined based on both macromorphology and micromorphology. Colony characteristics of the fungal strains grown on PDA, MEA, cornmeal agar (CMA), and oatmeal agar (OA; HiMedia®, HiMedia Laboratories Pvt. Ltd., Nashik, India) were observed after incubation in darkness at 25, 30, and 35 °C for 1–2 weeks, following previously described methods (Réblová et al. 2024). Micromorphological structures including conidiophores, conidiogenous cells, conidia, and other reproductive structures were examined using a light microscope (Nikon DS-Ri2; Nikon, Japan) from cultures grown on PDA at 25 °C for 30 days. At least 50 measurements were taken for each structure.

### DNA extraction, amplification, and sequencing

Fungal genomic DNA was extracted from fresh mycelia grown on PDA at 25 °C for one week using an E.Z.N.A.^®^ Tissue DNA Kit (Omega Bio-tek, USA). The internal transcribed spacer (ITS), large subunit (nrLSU), and small subunit (nrSSU) regions of the ribosomal RNA gene were amplified by polymerase chain reaction (PCR) using the primer pairs ITS5/ITS4, LR0R/LR5, and NS1/NS4, respectively (Vilgalys and Hester 1990; White et al. 1990). PCR amplifications were performed in 20 µL reaction mixtures containing 1 µL of genomic DNA, 1 µL of each primer, 10 µL of 2× Phanta Max Master Mix (Dye Plus) (Vazyme, China), and 7 µL of deionized water. Amplifications were conducted in a T100 Thermal Cycler (Bio-Rad, USA). The PCR conditions for ITS were as follows: initial denaturation at 95 °C for 2 min; 35 cycles of denaturation at 95 °C for 30 s, annealing at 52 °C for 30 s, and extension at 72 °C for 1 min; followed by a final extension at 72 °C for 10 min. The PCR conditions for LSU and SSU were: initial denaturation at 94 °C for 5 min; 35 cycles of denaturation at 94 °C for 30 s, annealing at 55 °C for 1 min, and extension at 72 °C for 2 min; followed by a final extension at 72 °C for 10 min. The quality and quantity of PCR products were assessed using 1% agarose gel electrophoresis and a NanoDrop 2000 spectrophotometer (Thermo Scientific, USA). PCR products of sufficient quality were sent to BGI-Shenzhen (Shenzhen, Guangdong, China) for sequencing.

### Phylogenetic analysis

Bidirectional sequence data were assembled using Sequencher v.5.4.6 (Nishimura 2000). The consensus sequences were compared with those in the National Center for Biotechnology Information (NCBI) database using the Basic Local Alignment Search Tool (BLAST; Altschul et al. 1990). Sequences used in the phylogenetic analyses were obtained from GenBank, and the accession numbers of all taxa included in this study are provided in Table 1. The sequence data for each locus were aligned separately using MAFFT (Katoh et al. 2009) and manually adjusted in BioEdit v.7.2.5 (Hall 2004). The three aligned loci were concatenated and formatted into a single alignment file using the ALTER online workflow (Glez-Peña et al. 2010). A concatenated dataset of LSU, ITS, and SSU sequences was used for the phylogenetic analysis of two novel taxa within Sordariomycetes. Maximum Likelihood (ML) and Bayesian Inference (BI) analyses were conducted to infer phylogenetic relationships. The ML analysis was performed using raxmlGUI v.1.3 (Silvestro and Michalak 2012) under the GTRGAMMAI substitution model, and branch support was estimated with 1,000 rapid bootstrap replicates. The optimal nucleotide substitution models for each individual locus were selected using MrModelTest v.2.3 based on the Akaike Information Criterion (AIC) (Nylander 2004). The GTR+I+G model was identified as the best-fitting substitution model for all loci. For the BI analysis, the concatenated alignment was prepared for MrBayes using PAUP v.3.99.169* (Swofford 2003) to embed the necessary block commands. Bayesian inference was performed using MrBayes v.3.2.4 (Ronquist et al. 2012) with Markov chain Monte Carlo (MCMC) sampling. Six simultaneous Markov chains were run for 1,000,000 generations, with trees sampled every 100 generations. The first 2,000 trees were discarded as burn-in, and the remaining 8,000 trees were used to calculate posterior probabilities (PP). The resulting phylogenetic trees were visualized using FigTree v.1.4.4 (Rambaut 2018). Branches were annotated only when they received bootstrap support (BS) ≥ 70% and posterior probabilities (PP) ≥ 0.90. Final graphical adjustments were made using Microsoft PowerPoint 2016.

**Table 1.** Details of sequences used in the molecular phylogenetic analysis.

Species	Strain/Isolate	GenBank accession number		
		LSU	ITS	SSU
*Afroraffaelea ambrosiae*	CBS 141678^T^	NG057115	–	NG061251
*Annulatascus thailandensis*	MFLUCC 18-1248^T^	NG_073783	NR171953	NG073526
*Annulatascus tratensis*	YJT22-1^T^	OP377977	OP377891	OP378052
*Aquimonospora tratensis*	MFLUCC 17-2133^T^	NG073661	MK335798	NG070637
*Ascitendus austriacus*	CBS 102665^T^	NG056942	–	NG061014
*Barbatosphaeria hippocrepida*	ICMP 17630^T^	NG058632	NR132087	NG065586
*Barbatosphaeria varioseptata*	CBS 137797^T^	NG058674	NR132089	NG065626
*Brachysporium polyseptatum*	DAOM 231136^T^	NG058624		
*Calyptosphaeria tenebrosa*	PRA 12740^T^	NG060434	NR158346	NG061261
*Ceratocystiopsis chalcographii*	CBS 147954^T^	OL309930	OL309930	–
*Ceratocystiopsis lunata*	CMW55897^T^	MW028141	MW028169	–
*Ceratocystiopsis minuta*	CBS 116796	EU913654	EU913695	–
*Ceratocystiopsis norroenii*	CBS 147956^T^	OL309936	NR182470	–
*Ceratocystiopsis quercina*	PC05.005^T^	JF909532	JF946755	JF909502
*Ceratosphaeria suthepensis* ^OUT^	PDD 76762^T^	NG079624	–	NG077399
*Ceratosphaeria yunnanensis* ^OUT^	KUMCC 21-0013^T^	NG149017	NR184381	–
*Ceratostomella crypta*	CBS 131683^T^	KM492871	KT991679	KM492860
*Ceratostomella cuspidata*	ICMP 17629	FJ617558	KT991671	KT991642
*Ceratostomella melanospora*	CBS 147993^T^	PQ215750	NR199102	NG244226
*Ceratostomella novae-zelandiae*	PDD 81433^T^	PQ215751	PQ215758	PQ215926
*Ceratostomella pyrenaica*	CBS 117116^T^	NG058735	NR199103	DQ076324
*Ceratostomella sordida*	CBS 116000^T^	NG060787	NR199104	AY761090
*Ceratostomella cuspidata*	IFBL 57.31	PQ215749	PQ215756	PQ215924
***Ceratostomella guangdongensis***	**MBSZU 26-007^T^**	**PZ159303**	**PZ160817**	**PZ167798**
***Ceratostomella guangdongensis***	**MBSZU 26-008**	**PZ159304**	**PZ160818**	**PZ167799**
***Ceratostomella guangdongensis***	**MBSZU 26-009**	**PZ159305**	**PZ160819**	**PZ167800**
*Conicotenuis fusiformis*	TSSK-1-1B	OR365482	OR365452	OR365489
*Diatrypasimilis australiensis*	ATCC MYA-3540^T^	FJ430581	NR111369	FJ430574
*Dictyosporella guizhouensis*	MFLU 18-1505^T^	NG073665	NR171836	NG073514
*Dictyosporella thailandensis*	MFLUCC 15-0985^T^	NG059175	NR152548	NG063645
*Distoseptispora amniculi*	MFLUCC 17-2129^T^	NG078696	NR174661	NG078768
*Distoseptispora effusa*	GZCC19_0532	MZ227224	MW133916	NG078754
*Distoseptispora muchuanensis*	CGMCC 3.27444^T^	NG244352	NR199255	PQ066585
*Distoseptispora phangngaensis*	MFLU 17-0855	MF077556	MF077545	MF077534
*Distoseptispora rayongensis*	MFLUCC 18-0415^T^	NG073624	NR171938	NG073504
*Fluminicola thailandensis*	MFLUCC 15-0984^T^	NG069505	NR153494	NG067659
*Fragosphaeria purpurea*	CBS 133.34	AB189154	OM501379	–
*Fragosphaeria reniformis*	CBS 134.34	AB189155	OM501381	–
*Graphilbum anningense*	CXY1939^T^	MH325162	MH555903	–
*Grosmannia aurea*	CBS 438.69^T^	MH871100	MH859345	AH007795
*Hypoxylon aurantium*	MFLU 16-1202^T^	NG068298	NR166287	NG068409
*Lanspora coronata*	AFTOL-ID 736	U46889	–	DQ470996
*Lanspora cylindrospora*	NFCCI4665	MN168891	MN168889	MN169053
*Lanspora dorisauae*	BCRC FU30316	OQ130043	OQ130045	OQ130044
*Lentomitella cirrhosa*	ICMP 15131^T^	NG059426	NR156322	NG064895
*Lentomitella magna*	ICMP 18371^T^	NG059798	NR156323	NG065106
*Lentomitella obscura*	CBS 138736^T^	NG058465	NR156324	NG065684
*Lentomitella tenuirostris*	CBS 138734^T^	NG058466	NR156327	NG063080
*Leptographium castellanum*	CBS 128697^T^	NG069991	NR156248	NG062839
*Lylea dalbergiae*	CBS 147004^T^	NG076731	NR173041	–
*Myrmecridium banksiae*	CBS 132536^T^	MH878335	NR111762	–
*Myrmecridium hydei*	MFLUCC 23-0217^T^	NG243371	NR190277	–
*Myrmecridium phragmiticola*	CPC 36367^T^	NG074444	NR170826	–
*Myrmecridium phragmitis*	CBS 131311^T^	NG057948	NR137782	–
*Neomyrmecridium asiaticum*	CBS 145080^T^	MK047494	NR161135	–
*Neomyrmecridium naviculare*	GZCC 20-0484^T^	NG243753	NR197498	NG242871
*Ophiostoma distortum*	CBS 429.82^T^	NG_067420	MH861510	–
*Ophiostoma floccosum*	CBS 799.73^T^	MH872536	MH860805	–
*Ophiostoma grandicarpum*	CBS 250.88^T^	MH873820	MH862127	–
*Ophiostoma multiannulatum*	CMW2567^T^	DQ294366	FJ959049	–
*Ophiostoma piliferum*	CBS 129.32	AY281094	–	AJ243295
*Ophiostoma pulvinisporum*	CMW9022^T^	DQ294380	AY546714	–
*Ophiostoma rachisporum*	CBS 128119^T^	NG067467	MH864858	–
*Ophiostoma subannulatum*	CMW518^T^	DQ294364	AY934522	–
*Pararamichloridium aquisubtropicum*	GZCC 21-0668^T^	NG228929	NR185675	–
*Pararamichloridium caricicola*	CBS 145069^T^	MK047488	MK047438	–
*Pararamichloridium livistonae*	CBS 144522	MK442542	MK442606	–
*Pararamichloridium ouropretoense*	COAD 3991^T^	NG245764	NR201082	–
***Pararamichloridium purpureum***	**MBSZU 26-006^T^**	**PZ159302**	**PZ160816**	**PZ167797**
***Pararamichloridium purpureum***	**MBSZU 26-109**	**PZ373967**	**PZ373965**	**PZ373966**
*Pararamichloridium verrucosum*	CBS 128.86^T^	NG057768	NR156653	NG065540
*Phomatospora bellaminuta*	AFTOL-ID 766	FJ176857	–	FJ176803
*Phomatospora biseriata*	MFLUCC 14-0832A	KX549448	KX549453	KX549458
*Raffaelea canadensis*	CBS 168.66^T^	EU984299	GQ225699	–
*Raffaelea crossotarsa*	Hulcr7182^T^	KX267103	KX267135	–
*Raffaelea sulcati*	CBS 806.70^T^	NG_064084	MH859951	NG062681
*Raffaelea tritirachium*	CBS 726.69	MH871169	NR_160119	NG063092
*Rhamphoriopsis aquimicrospora*	GZCC 20-0515^T^	NG243748	NR197493	NG242864
*Sporidesmium tratense*	MFLUCC 17-2392^T^	NG243764	NR197512	NG242885
*Wongia fusiformis*	MFLUCC 21-0028^T^	NG077381	NR174651	NG077449
*Xenoanthostomella chromolaenae*	MFLUCC 17-1484^T^	NG068915	NR168230	NG068421
*Xylolentia aseptata*	GZCC 20-0424^T^	NG243756	NR197502	NG242873
*Xylolentia brunneola*	PRA-13611	MG600398	NR160343	MG600407

Fungal species obtained in this study are bold. Superscript “T” represents ex-type species. Superscript “OUT” represents the outgroup taxa. “–” represents the absence of sequence data in GenBank database.

## Results

### Fungal isolation

A total of five fungal strains (LBP1, LBP2, GD065, GD090, and GD091) were obtained in this study. Two strains (LBP1 and LBP2) were obtained from air contaminants, whereas the remaining three strains (GD065, GD090, and GD091) were isolated from rock surfaces. All strains were deposited in the Culture Collection of MBSZU under the accession numbers MBSZU 26-006, MBSZU 26-109, MBSZU 26-007, MBSZU 26-008, and MBSZU 26-009, respectively.

### Phylogenetic analysis

**Figure 1.**
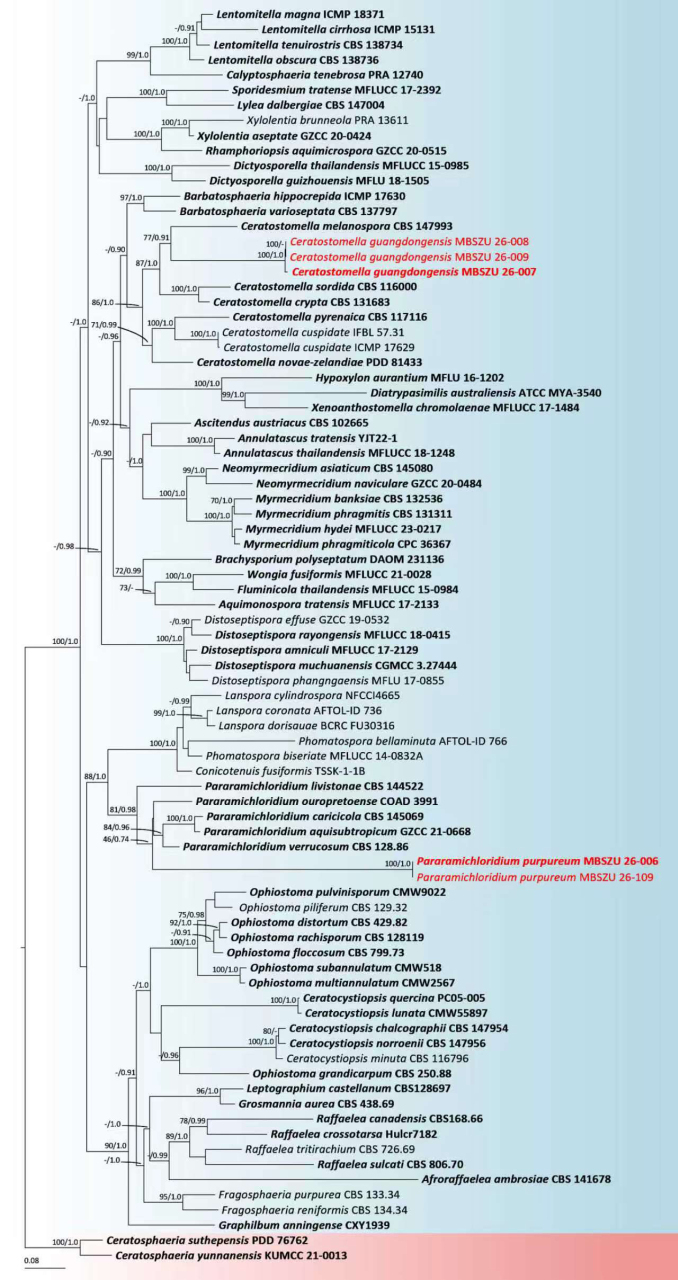
Phylogram generated from maximum likelihood analysis of a combined LSU, ITS, and SSU genes of 83 sequences. *Ceratosphaeria suthepensis* (PDD 76762) and *C. yunnanensis* (KUMCC 21-0013) were used as outgroups. Bootstrap values ≥ 70% ML (left) and Bayesian posterior probabilities ≥ 0.90 (right) are shown above nodes. Lower support values are also shown for key nodes of interest. The scale bar represents the expected number of nucleotide substitutions per site. The sequences of fungal species obtained in this study are in red. Type species are in bold.

Phylogenetic analysis of 83 taxa representing the major lineages of Sordariomycetes was performed using a combined LSU, ITS, and SSU sequence dataset. The aligned matrix comprised 3,826 characters including gaps (LSU: 1–1,070, ITS: 1,071–2,129, SSU: 2,130–3,826). The best-scoring RAxML tree had a final ML optimization likelihood value of −37836.85. The matrix contained 1,803 distinct alignment patterns with 44.2091% undetermined characters or gaps. The estimated base frequencies were A = 0.249050, C = 0.242863, G = 0.284403, and T = 0.223683; substitution rates were AC = 1.394060, AG = 1.871576, AT = 1.253509, CG = 1.269107, CT = 4.665867, and GT = 1.0000. The gamma distribution shape parameter (α) was estimated as 0.427407, and the tree length was 10.388406. In the Bayesian inference (BI) analysis, the final average standard deviation of split frequencies after the MCMC generations was 0.007384. The ML and BI analyses resulted in similar topologies; therefore, the ML tree is presented in Fig. 1. The phylogenetic tree revealed that the five strains obtained in this study formed two distinct lineages within Sordariomycetes. Three rock-inhabiting strains (MBSZU 26-007, MBSZU 26-008, and MBSZU 26-009) clustered within the genus *Ceratostomella* and formed a well-supported monophyletic lineage distinct from previously described species (100% BS, 1.00 PP). The air-contaminated strains (MBSZU 26-006 and MBSZU 26-109) were placed within the genus *Pararamichloridium*, forming a basal lineage to *P. aquisubtropicum*, *P. caricicola*, *P. ouropretoense*, and *P. verrucosum*, although this relationship received relatively low statistical support (46% BS, 0.74 PP).

### Taxonomy

#### Ceratostomella guangdongensis

Taxon classificationFungiBolinialesBoliniaceae

W.Q. Meng, T. Thitla, S. Hongsanan sp. nov.

25E9199E-4852-5488-9875-88258EC06712

863235

Fig. 2

##### Etymology.

The specific epithet *guangdongensis* refers to Guangdong Province, China, where the holotype was collected.

**Figure 2.**
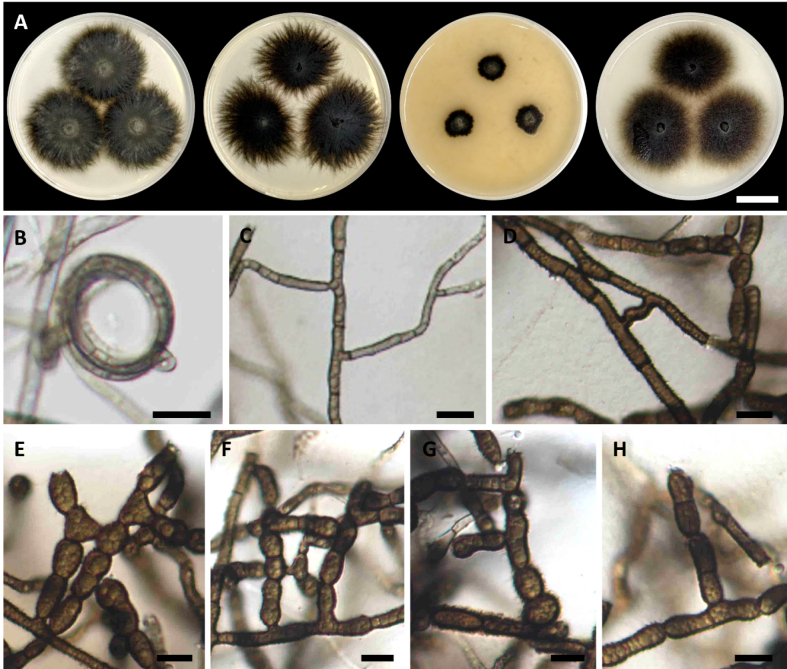
*Ceratostomella guangdongensis* (MBSZU 26-007, ex-type living culture). **A**. Colony morphology at 30 °C after 10 days on PDA, MEA, OA, and CMA (from left to right); **B–D**. Vegetative hyphae with hyphal coil and anastomoses; **E–H**. Monilioid hyphae with 0–1 septa and varying shapes. Scale bars: 2 cm (**A**); 10 µm (**B–H**).

##### Type.

China • Guangdong Province, Shenzhen, Dananshan Mountain (22°30'06.7"N, 113°54'44.0"E), on rock sampled from the mountain, 25 March 2025, W.Q. Meng & T. Thitla; GD065 (SZU26-166, holotype), ex-type living culture MBSZU 26-007, dried culture permanently preserved in a metabolically inactive state, SZU26-166.

##### Colony diam.

(in mm) 7 days, 25 °C: PDA 42–45, MEA 43–49, OA 12–17. 30 °C: PDA 44–46, MEA 50–55, OA 18–20. 35 °C: PDA 46–48, MEA 50–55, OA 18–21.

##### Culture characteristics.

Colonies were black with a black reverse on all media. On PDA, colonies were circular, spreading, and flat, covered with a thin white downy mycelium, with an irregular filamentous margin. On MEA, colonies were circular, spreading, and flat, with a filamentous margin. On OA, slow growth rate, colonies were circular, flat, smooth-textured, and restricted, with an entire margin. On CMA, colonies were circular, spreading, flat, and smooth-textured, with a filamentous margin. Soluble pigments were not produced on any medium, and sporulation was absent on all culture media.

##### Micromorphology.

Mycelium composed of submerged, smooth, septate, branched, thin- to thick-walled, subhyaline to pale brown hyphae, 1.5–4.5 µm (x̄ = 2.7 ± 0.6 µm, n = 50). Hyphae frequently convert into monilioid hyphae consisting of rough, thick-walled, 0–1 septate, guttulate cells that are brown to dark brown, varying in shape from subglobose to cylindrical 4–8 × 8–23 µm (x̄ = 6 ± 0.8 × 14.5 ± 3.8 µm, n = 50). Branching of the monilioid hyphae commonly occurs at right angles. Hyphal coils and anastomoses are also present.

##### Additional strain examined.

China • Guangdong Province, Shenzhen, Dananshan Mountain (22°30'06.7"N, 113°54'44.0"E), on rock sampled from the mountain, 25 March 2025, W.Q. Meng & T. Thitla; GD090 and GD091, living culture MBSZU 26-008, and MBSZU 26-009, permanently preserved in a metabolically inactive state.

##### Habitat and distribution.

On rock; currently known only from Shenzhen, Guangdong Province, China.

##### Notes.

Phylogenetic analyses based on the combined LSU, ITS, and SSU sequence dataset placed the three new strains within the *Ceratostomella* clade (Fig. 1), forming a sister lineage to *C. melanospora* with 72% bootstrap support and a posterior probability of 0.91. However, the new strains differ morphologically from *C. melanospora* in a combination of characters, including the presence of monilioid hyphae, which have not been reported in *C. melanospora* (Réblová et al. 2024). Moreover, the new species exhibits relatively high temperature tolerance, with optimal growth observed at 35 °C, whereas *C. melanospora* does not grow at this temperature. Ecologically, *C. melanospora* has been reported as a saprobe on decaying wood, whereas the strains obtained in this study were isolated from rock (Réblová et al. 2024). Pairwise nucleotide comparisons between *C. guangdongensis* and *C. melanospora* revealed sequence differences of 23.34% in the ITS region (116/497 bp, excluding gaps), 7.66% in the LSU region (65/849 bp, excluding gaps), and 1.85% in the SSU region (18/973 bp, excluding gaps). Based on morphological characteristics, growth temperature, ecological differences, phylogenetic evidence, and pairwise nucleotide comparisons, we introduce *Ceratostomella guangdongensis* as a novel species of *Ceratostomella*.

#### Pararamichloridium purpureum

Taxon classificationFungiPararamichloridialesPararamichloridiaceae

W.Q. Meng, S. Khuna, S. Hongsanan sp. nov.

A23AE7A4-6D07-53B9-96C9-552C54F76349

863236

Fig. 3

##### Etymology.

The specific epithet *purpureum* refers to the diagnostic purple diffusible pigment produced by the colony in culture.

**Figure 3.**
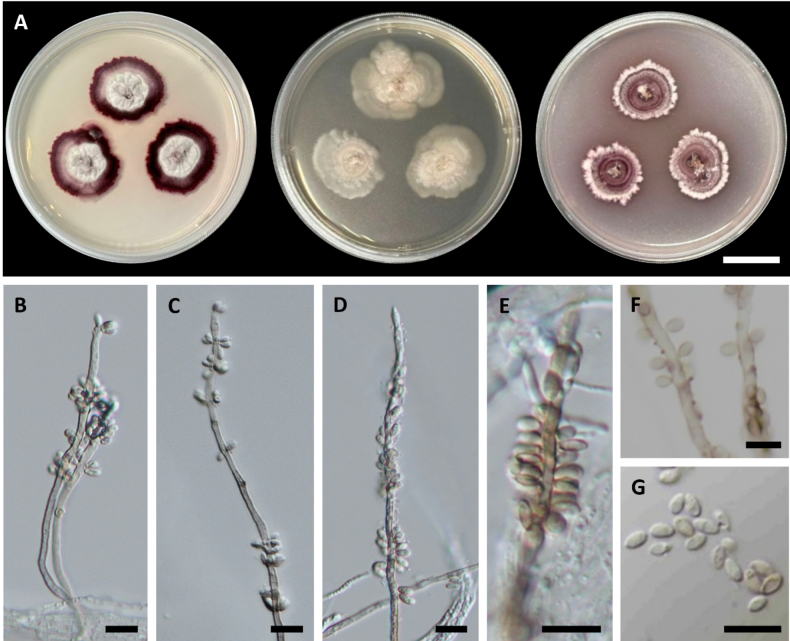
*Pararamichloridium purpureum* (MBSZU 25-006, ex-type living culture). **A**. Colony morphology at 25 °C after 2 weeks on PDA, MEA, and CMA (from left to right); **B–E**. Conidiophores, conidiogenous cells, and conidia; **F**. Rachis with pimple-like denticles; **G**. Conidia. Scale bars: 2 cm (**A**); 10 µm (**B–G**).

##### Type.

China • Guangdong Province, Shenzhen, Shenzhen University, College of Life Sciences and Oceanography (22°35'55.0"N, 113°59'21.0"E), from air, 10 July 2025, W.Q. Meng; LBP1 (SZU26-165, holotype); ex-type living culture MBSZU 25-006, dried culture permanently preserved in a metabolically inactive state, SZU26-165.

##### Colony diam.

(in mm) 14 days, 25 °C: PDA 34–36, MEA 26–29, CMA 34–39; 30 °C: PDA 13–15, MEA 10–12, CMA 15-18; 35 °C: no or negligible growth on all media.

##### Culture characteristics.

Colonies on PDA were circular, restricted, centrally raised, and wrinkled, with a white surface and a violet-red, slightly undulate margin. The reverse was dark yellow at the center and violet-red toward the margin. Colonies on MEA were irregular, flat, restricted, and wrinkled, pale yellow at the center with a white, entire margin. The reverse was pale yellow with a white margin. Colonies on CMA were circular and raised, grayish-violet at the center, with a white, slightly raised, and undulate margin. The reverse was deep violet-red to white. A soluble violet-red pigment diffusing into the surrounding medium was observed around colonies on PDA and CMA. Sporulation was abundant on all media.

##### Micromorphology.

Mycelium consisting of septate, branched, smooth, hyaline hyphae, 1.2–2.1 µm (x̄ = 1.67 ± 0.24 µm, n = 50) diam. Conidiophores macronematous, solitary, unbranched, septate, erect, straight to flexuous, arising from superficial hyphae, cylindrical, pale brown to brown, 30.4–104.4 × 1.5–3.2 µm (x̄ = 65.0 ± 20.3 × 2.3 ± 0.4 µm, n = 50). Conidiogenous cells integrated, terminal, holoblastic, polyblastic, subhyaline to pale brown, cylindrical, 41.5–203.0 × 1.5–2.8 µm (x̄ = 111.5 ± 42.1 × 2.2 ± 0.4 µm, n = 50), sympodially proliferate, forming a rachis with numerous pimple-like denticles, inconspicuous, slightly darkened not thickened. Conidia solitary, smooth-walled, aseptate, hyaline to pale brown, ellipsoidal, 3.6–5.6 × 2.2–3.5 µm (x̄ = 4.6 ± 0.6 × 2.7 ± 0.4 µm, n = 50). Sexual morph not observed.

##### Habitat and distribution.

Air; known only from Shenzhen, Guangdong Province, China.

##### Notes.

*Pararamichloridium*, the type genus of Pararamichloridiaceae, was established by Crous et al. (2017), with *P. livistonae* designated as the type species, originally isolated from leaves of *Livistona australis* collected in Australia (Crous et al. 2017). The genus currently comprises five species, namely *P. aquisubtropicum*, *P. caricicola*, *P. livistonae*, *P. ouropretoense*, and *P. verrucosum* (Crous et al. 2025).

In this study, we introduce *Pararamichloridium purpureum* as the sixth species of the genus, isolated from laboratory air in Shenzhen, Guangdong Province, China. The species is proposed based on morphological characteristics and multigene phylogenetic analyses inferred from a combined dataset of LSU, ITS, and SSU sequences. Phylogenetically, the new species forms a distinct lineage within *Pararamichloridium* and is closely related to *P. aquisubtropicum*, *P. caricicola*, *P. ouropretoense*, and *P. verrucosum* (Fig. 1). However, the new strain forms an independent branch within the genus and is clearly distinguishable from related species based on both morphological and molecular evidence.

Morphologically, the new species differs from *P. aquisubtropicum*, which produces relatively long conidiophores (119–202 µm) and larger conidia (4–8 × 3–4.7 μm) (Jayawardena et al. 2023), whereas *P. purpureum* produces shorter conidiophores (30.4–104.4 × 1.5–3.2 µm) and smaller conidia (3.4–5.6 × 2.2–3.5 µm). The new species can also be distinguished from *P. caricicola*, which produces relatively slender conidia measuring 6–7 × 3–3.5 µm, whereas *P. purpureum* forms comparatively ellipsoidal conidia (Crous et al. 2018). In addition, *P. verrucosum* is characterized by verrucose conidia, a feature not observed in *P. purpureum* (Crous et al. 2017). *Pararamichloridium ouropretoense* differs from the new species in having larger conidia (6–8 × 3–4 µm) and more robust conidiophores (50–150 × 4–5 µm) (Crous et al. 2025).

Pairwise nucleotide comparisons further support the distinctiveness of the new species. The ITS sequence of *P. purpureum* differs from those of *P. aquisubtropicum*, *P. caricicola*, *P. ouropretoense*, and *P. verrucosum* by 121/519 bp (23.31%), 131/561 bp (23.35%), 132/538 bp (24.54%), and 139/528 bp (26.33%), respectively (excluding gaps). Similarly, LSU sequence comparisons revealed differences of 100/706 bp (14.16%), 99/704 bp (14.06%), 94/703 bp (13.37%), and 92/704 bp (13.07%) between the new species and these taxa. SSU sequence comparison was only possible with *P. verrucosum*, as SSU sequence data are not available for the other related species, and the sequences differed 45/833 bp (5.40%). Ecologically, *P. purpureum* was isolated from air contaminants collected using exposed culture plates, whereas previously described species of *Pararamichloridium* have mainly been reported from plant-associated substrates or submerged environments. The new strain also consistently produces a diffusible violet-purple pigment on PDA.

Taken together, phylogenetic placement, morphological differences, nucleotide divergence, and cultural characteristics support the recognition of the strain as a novel species of *Pararamichloridium*.

## Discussion

The class Sordariomycetes is one of the largest and most phylogenetically diverse groups within the Ascomycota, comprising fungi with diverse lifestyles, including saprobes, endophytes, pathogens, and species capable of tolerating extreme environmental conditions. Members of this class occur in virtually all environments, including plant debris, soil, freshwater habitats, and even bare rock surfaces (Zhang and Wang 2015). In recent years, the increasing application of molecular tools in fungal taxonomy has led to the discovery and description of numerous new taxa within this class (Wang et al. 2023).

In this study, LSU, ITS, and SSU sequence data were generated and used to infer the phylogenetic placement of two fungal species collected from Guangdong Province, China. Phylogenetic analyses indicated that one species belongs to *Ceratostomella* (Barbatosphaeriaceae, Barbatosphaeriales), whereas the other is affiliated with *Pararamichloridium* (Pararamichloridiaceae, Pararamichloridiales). The *Ceratostomella* strains formed a sister lineage to *C. melanospora*; however, morphological and molecular evidence clearly distinguish them as a separate species. The second strain formed an independent lineage within *Pararamichloridium*, clearly distinct from previously described species based on morphological characteristics, ecological traits, and nucleotide comparisons. Accordingly, we introduce these strains as two novel species of Sordariomycetes, namely *Ceratostomella guangdongensis* sp. nov. and *Pararamichloridium purpureum* sp. nov.

Species of the genus *Ceratostomella* are commonly associated with plant-derived substrates, particularly decaying wood and other lignocellulosic materials, where they occur as saprotrophs (Réblová et al. 2024). In the present study, *Ceratostomella guangdongensis* was isolated from a rock surface and recovered as a distinct lineage closely related to *C. melanospora* in the phylogenetic analyses. Although the two taxa are phylogenetically related, *C. guangdongensis* can be distinguished from *C. melanospora* and other species described by a combination of cultural characteristics, growth at 35 °C, and sequence differences in the analyzed loci. The isolation of this species from a rock substrate also expands the currently known substrate associations within the genus.

No sexual or asexual sporulation was observed in *C. guangdongensis* despite repeated cultivation attempts on different media under various incubation conditions. The absence of reproductive structures limits morphological characterization, as species of *Ceratostomella* are traditionally distinguished based on ascomatal and ascospore features. Therefore, the taxonomic placement of this species relies primarily on multi-locus phylogenetic evidence together with vegetative characteristics. Future collections containing fertile material will be important for confirming the morphological circumscription and taxonomic placement of this species within *Ceratostomella*. Taken together, the molecular, physiological, and cultural characteristics support the recognition of *C. guangdongensis* as a distinct species within the genus.

Species of the genus *Pararamichloridium* have previously been reported mainly from plant-associated substrates and freshwater environments (Crous et al. 2017; Luo et al. 2019). In the present study, the new species was isolated from indoor air using a culture-dependent sampling approach, representing a new ecological niche for the genus. In addition, the new species consistently produces a diffusible violet-purple pigment on common culture media, a feature that has not been reported in closely related species (Després et al. 2012; Peay et al. 2016). Morphological comparisons also revealed differences in conidial shape and size compared with related taxa such as *P. caricicola*, which possesses more elongated conidia (Crous et al. 2017).

Phylogenetic inference in this study was based primarily on ITS and LSU datasets, as SSU sequences are currently unavailable for *P. aquisubtropicum*, *P. caricicola*, *P. ouropretoense*, and several other related species within the genus *Pararamichloridium*. The absence of SSU data represents a limitation of the present study, as it may reduce the robustness and deeper resolution of the multi-locus phylogeny. Future taxonomic studies of this genus would benefit from the inclusion of SSU sequence data to enable more comprehensive phylogenetic analyses.

Overall, the discovery of these two novel species expands the known morphological, ecological, and physiological diversity of these genera within the class Sordariomycetes (Réblová et al. 2024). These findings further emphasize that understudied environments, such as rock surfaces and air habitats, may represent important reservoirs of undiscovered fungal diversity (Peay et al. 2016; Coleine et al. 2021). Continued exploration of such habitats, together with integrative taxonomic approaches combining morphology and multi-locus phylogenetic analyses, will contribute significantly to a more comprehensive understanding of fungal diversity and evolution within Sordariomycetes.

## Supplementary Material

XML Treatment for Ceratostomella guangdongensis

XML Treatment for Pararamichloridium purpureum

## Additional information

### Artificial Intelligence (AI) use

The authors accept full responsibility for the content of the manuscript, including the disclosure of any use of AI.

No AI tools were used in the preparation of this manuscript.

### Author ORCIDs

W. Meng https://orcid.org/0009-0006-3840-0992

S. Khuna https://orcid.org/0000-0003-1999-4001

T. Thitla https://orcid.org/0000-0003-3092-0679

N. Xie https://orcid.org/0000-0002-5866-8535

S. Hongsanan https://orcid.org/0000-0003-0550-3152

### Data availability

All of the data that support the findings of this study are available in the main text.
